# Supplementary material for: Reduction of SEM charging artefacts in native cryogenic biological samples
Source: Nat Commun. 2025 Jun 4;16:5204. doi: 10.1038/s41467-025-60545-3 (PMC12137866; doi:10.1038/s41467-025-60545-3)
Supplement: Supplementary file 1 — Supplementary Information [file 41467_2025_60545_MOESM1_ESM.pdf]

Supplementary information:

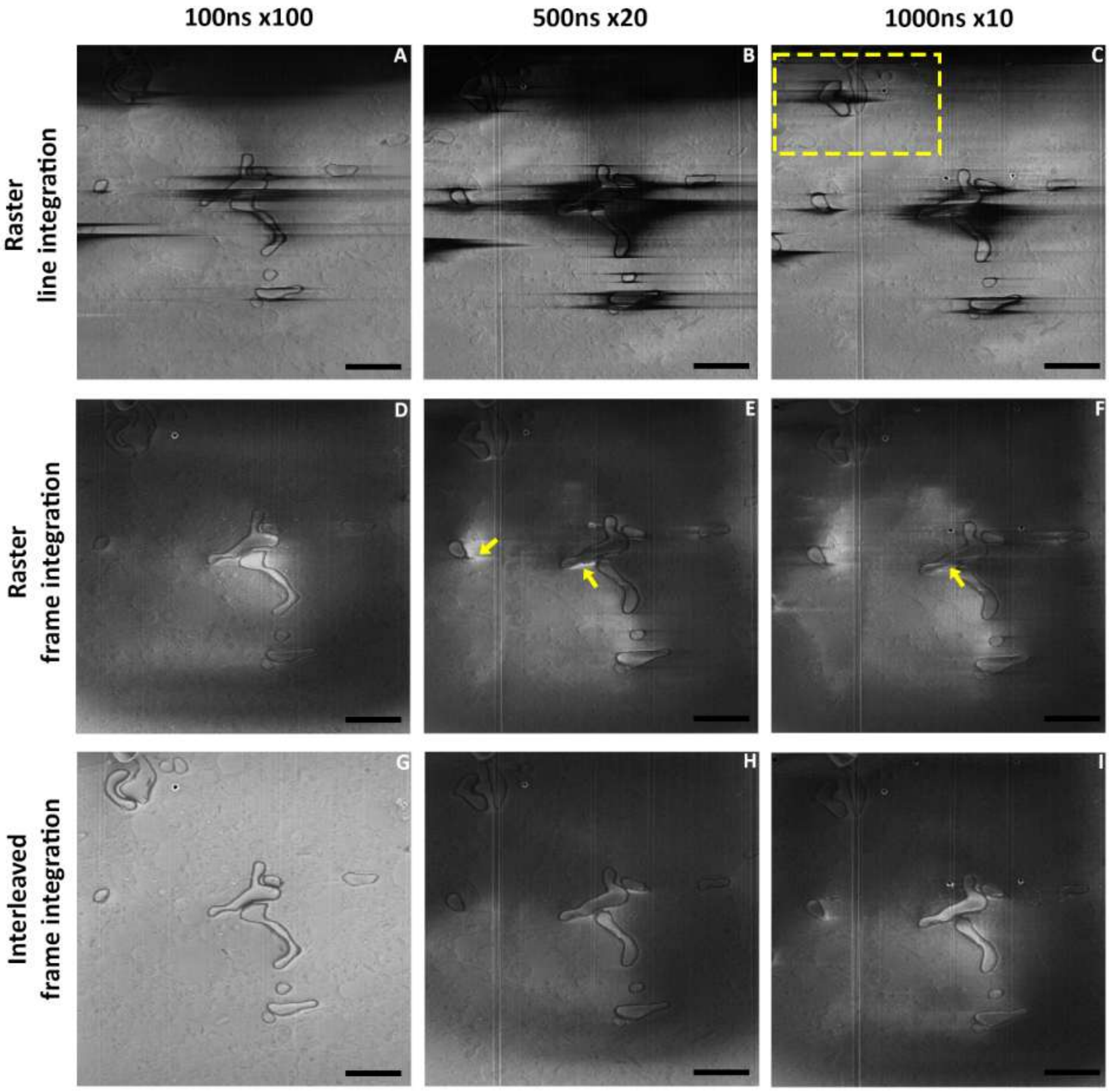

Supplementary Figure 1: Effect of electron fluence distribution on charging artefacts (SEM imaging at 52º angle to the FIB milled sample plane).

(A-I) Representative images of brain tissue for different pixel fluences and raster or interleaved scan patterns with (G) interleaved frame integration at 100 ns x 100 showing the greatest improvement in charging artifacts. Arrows: bright charging artefacts. Dashed rectangle: region where the longer dwell allowed the observation of biological features. Scale bar: 2 µm.

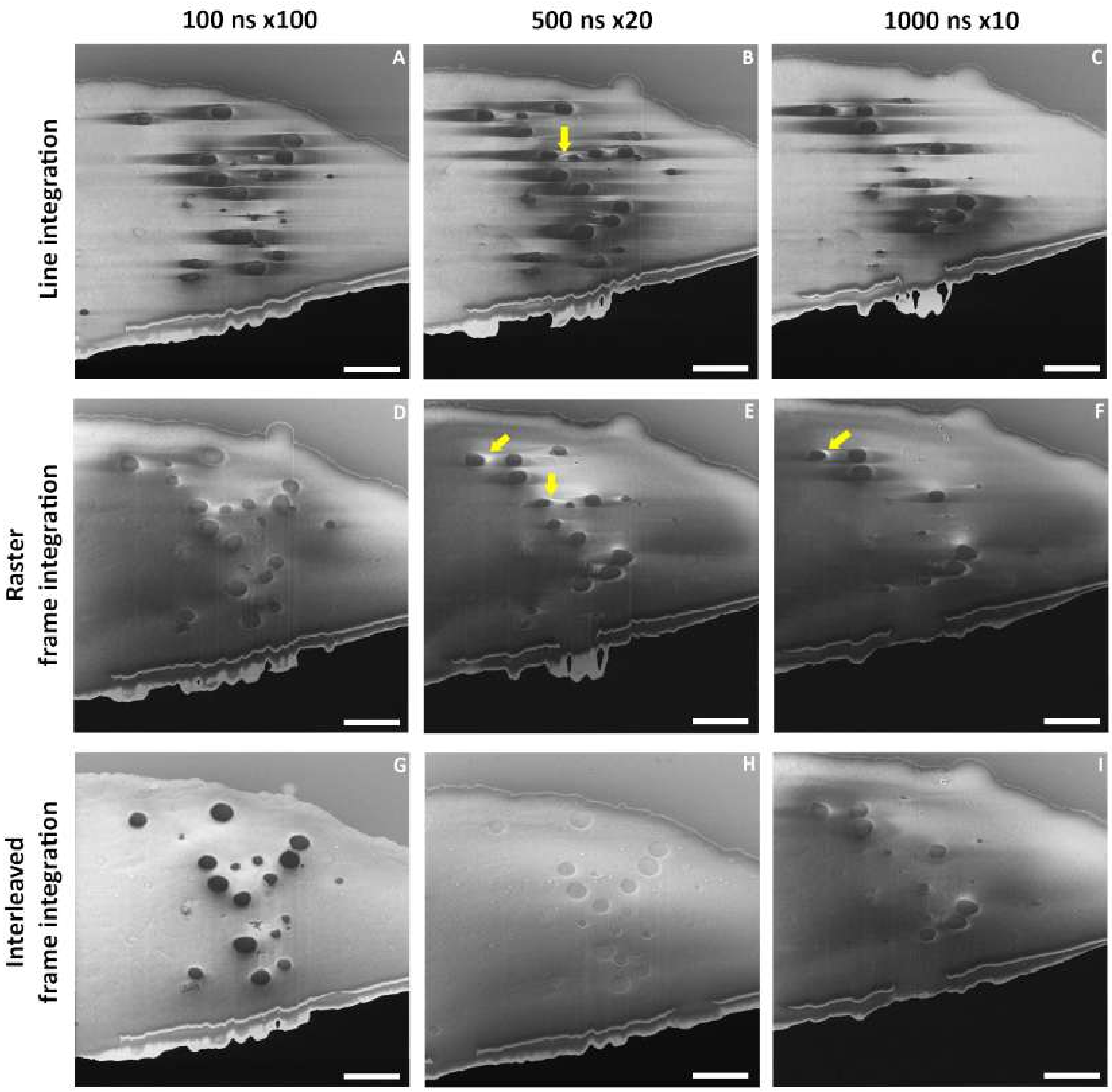

Supplementary Figure 2: Effect of electron fluence distribution on charging artefacts.
Vitrified RPE-1 cells imaged at 52° with respect to the FIB milled sample plane using the same parameters for dwell time and number of repetitions (for frame or line integration) but different scanning strategies. To avoid accumulation of potential beam damage, the area was milled between image acquisitions (50 nm steps). Arrows indicate the presence of bright charging artefacts. Scale bar: 2 µm.

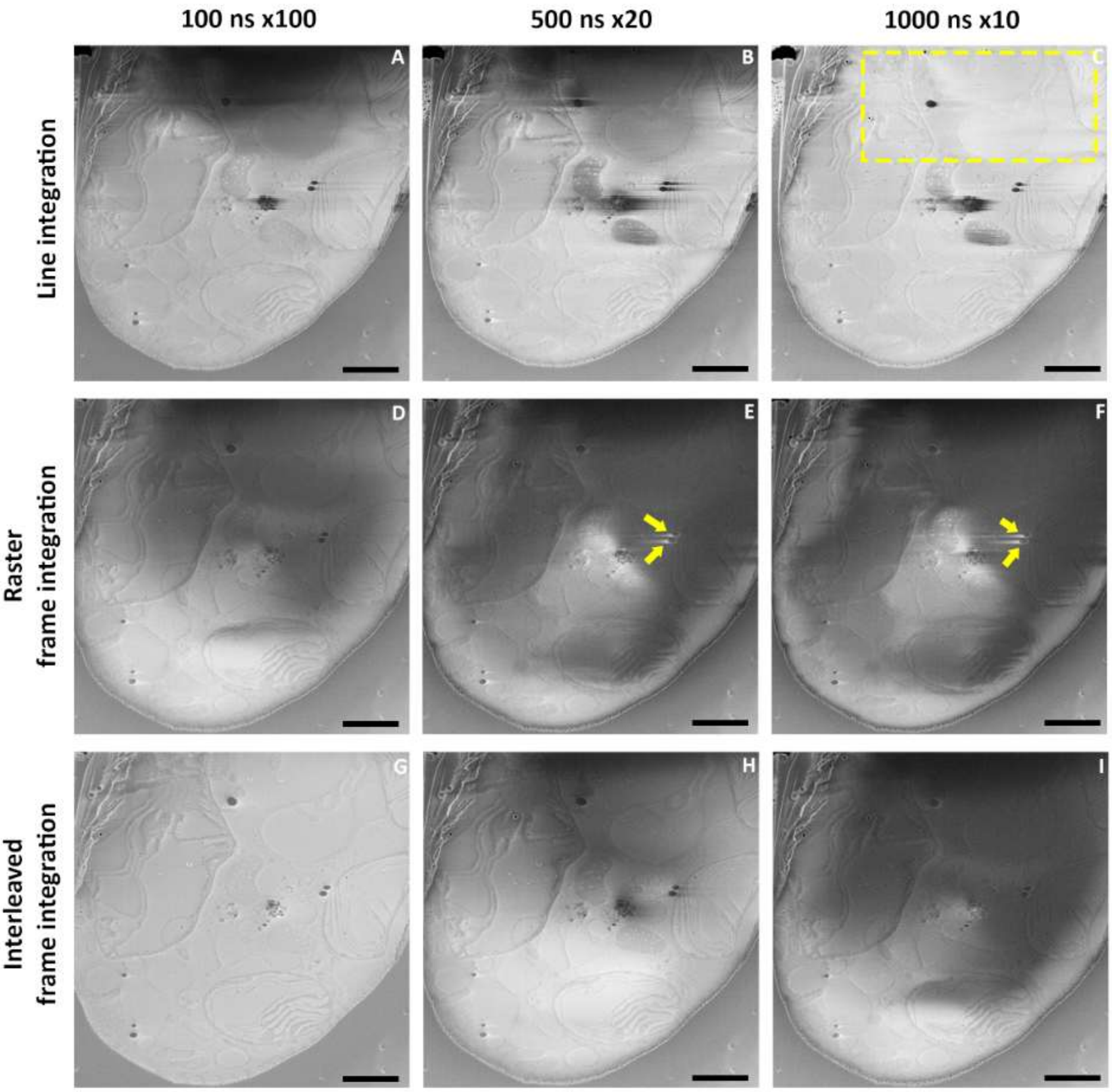

Supplementary Figure 3: Effect of electron fluence distribution on charging artefacts.
Vitrified *E. gracilis* imaged at 52° with respect to the FIB milled sample plane using the same parameters for dwell time and number of repetitions (for frame or line integration) but different scanning strategies. To avoid accumulation of potential beam damage, the area was milled between image acquisitions (50 nm steps). Arrows indicate the presence of bright charging artefacts. Dashed rectangle: region where the longer dwell allowed the observation of biological features. Scale bar: 2 µm.

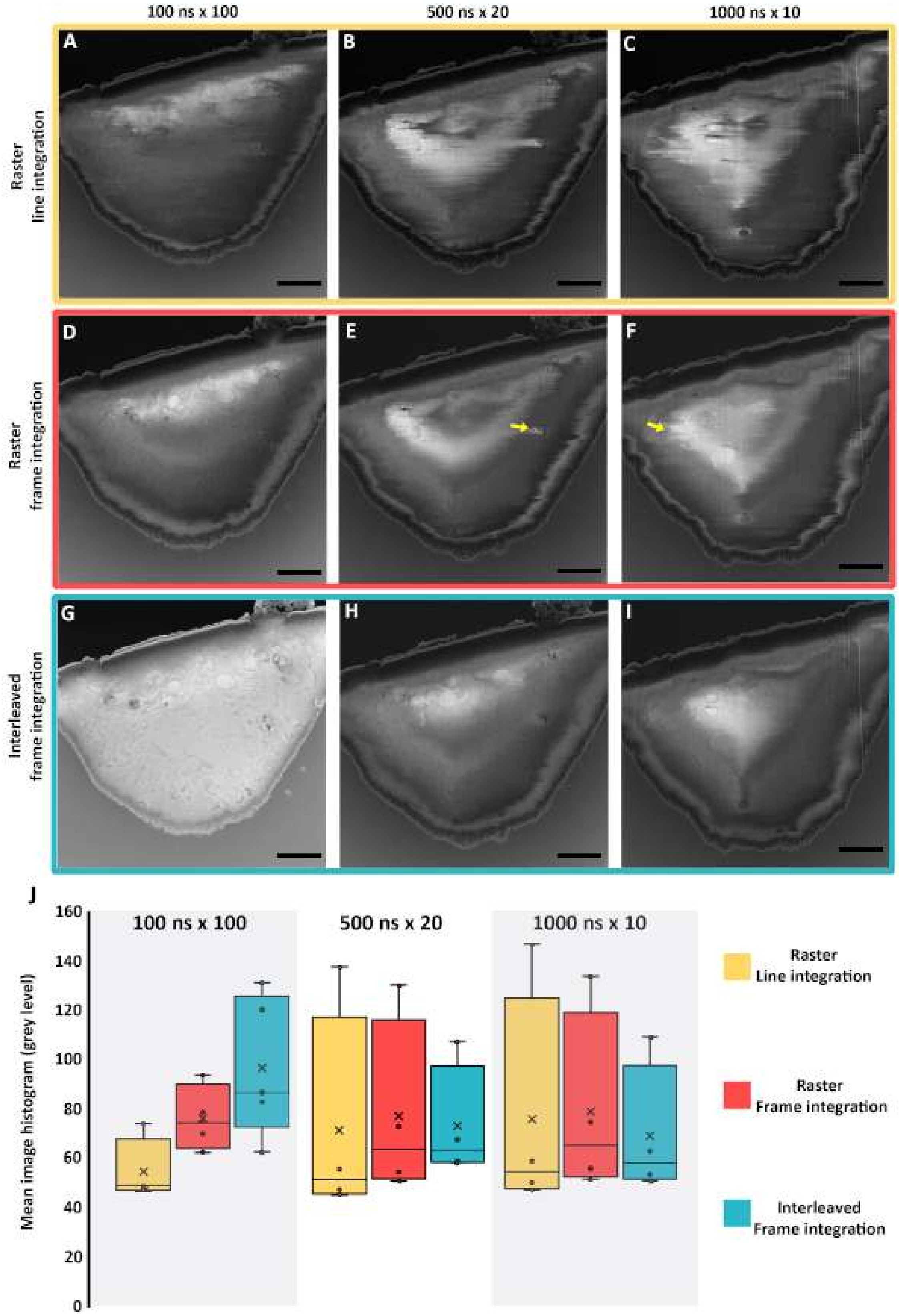
100 ns x 100
500 ns x 20
1000 ns x 10
Raster
line integration
Raster
frame integration
Interleaved
frame integration
A
B
C
D
E
F
G
H
I
J
Mean image histogram (grey level)
Raster
Line integration
Raster
Frame integration
Interleaved
Frame integration

Supplementary Figure 4: Effect of electron fluence distribution on charging artefacts (SEM imaging perpendicular to the FIB milled sample plane).

(A-I) Images of RPE-1 cell for different pixel fluence strategies and raster or interleaved scan patterns with (G) interleaved frame integration at short 100 ns x 100 showing the greatest reduction in charging artifacts. Arrows: bright charging artefacts. Dashed rectangle region recorded with a with higher fluence. Scale bar: 2 µm. (J): Mean of image histograms for different populations (n>4) from vitrified RPE-1, *E.gracilis* and mouse brain represented as a circle. Population median (middle line), population mean (cross), median of the 1st quartile of the population (bottom line of the box), median of the 3rd quartile of the population (top line of the box). Vertical lines extend to minimum and maximum values. Statistical analysis is given in Supplementary Table 2. Images acquired using interleaved scanning with frame integration associated with short dwell time and high integration (100 ns x100) form a dataset for which the mean of the histogram is at 97, close to 127 which is the mid-range histogram intensity value of unsigned 8-bit images.

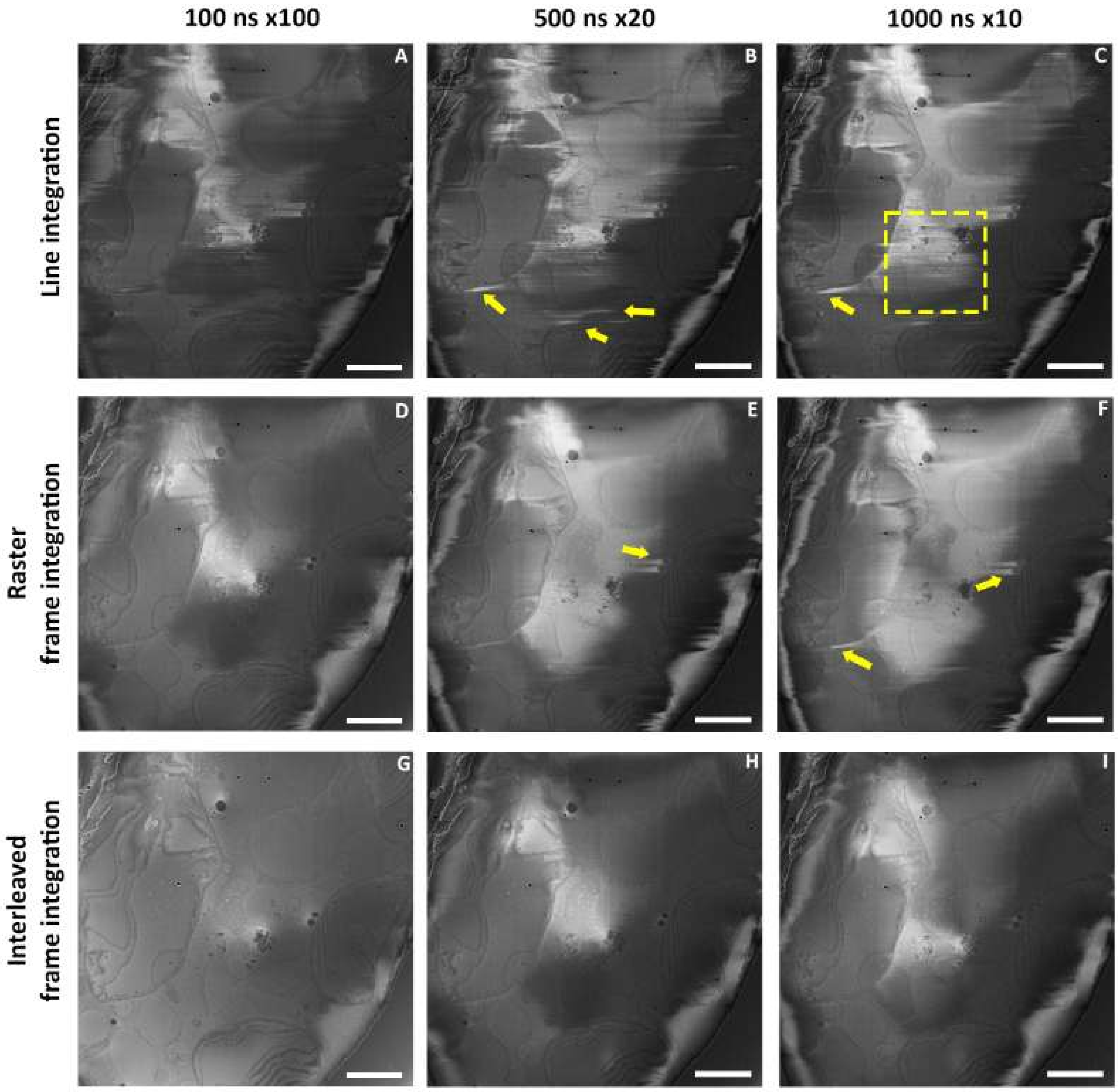

Supplementary Figure 5: Effect of electron fluence distribution on charging artefacts.
Vitrified *E. gracilis* imaged at 90° with respect to the FIB milled sample plane using the same parameters of dwell time and number of repetitions (for frame or line integration) but different scanning strategies. To avoid accumulation of potential beam damage, the area is milled between image acquisitions (50 nm steps). Dashed rectangle: region where the longer dwell allowed the observation of biological features. Arrows: bright charging artefacts. Scale bar: 2 μm.

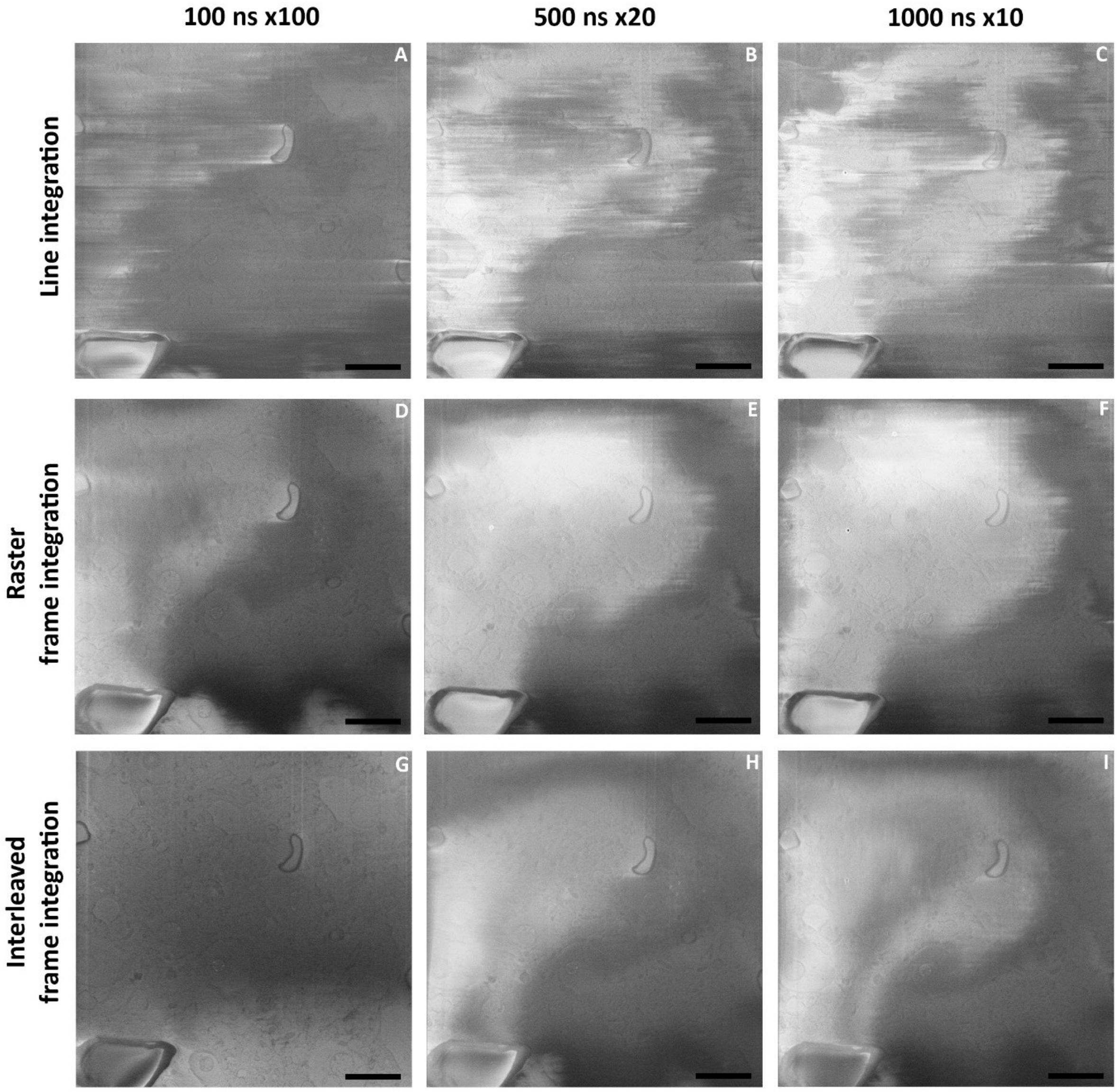

Supplementary Figure 6: Effect of electron fluence distribution on charging artefacts.
Vitrified 118-days old mouse brain slice imaged at 90° with respect to the FIB milled sample plane using the same parameters of dwell time and number of repetitions (for frame or line integration) but different scanning strategies. To avoid accumulation of potential beam damage, the area was milled between image acquisitions (50 nm steps). Scale bar: 2 µm.

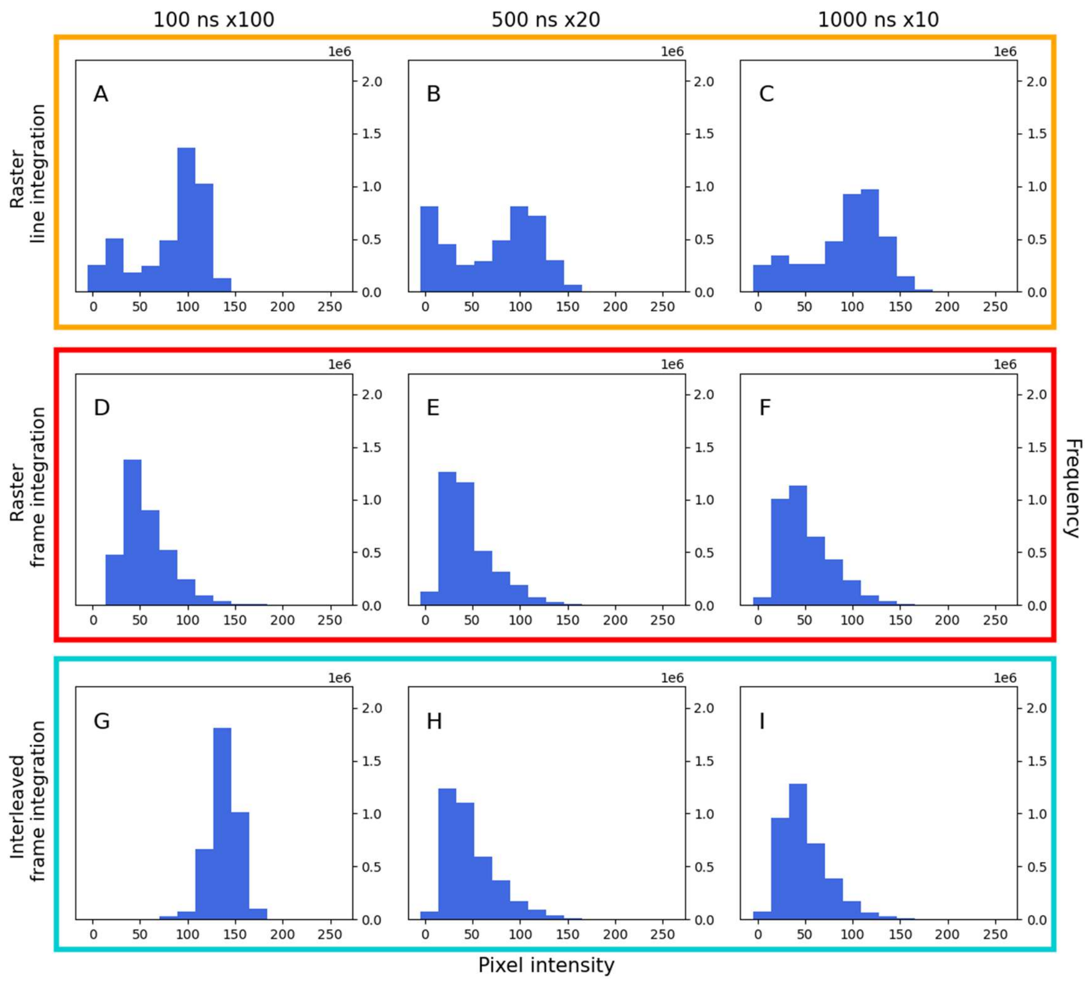

Supplementary Figure 7: Histograms from images of brain tissue acquired at different electron fluence.
(A-I) Intensity histograms calculated from representative images of brain tissue for different pixel fluence strategies and raster or interleaved scan patterns. To avoid accumulation of surface beam damage, the area was FIB milled between image acquisitions by 50 nm.

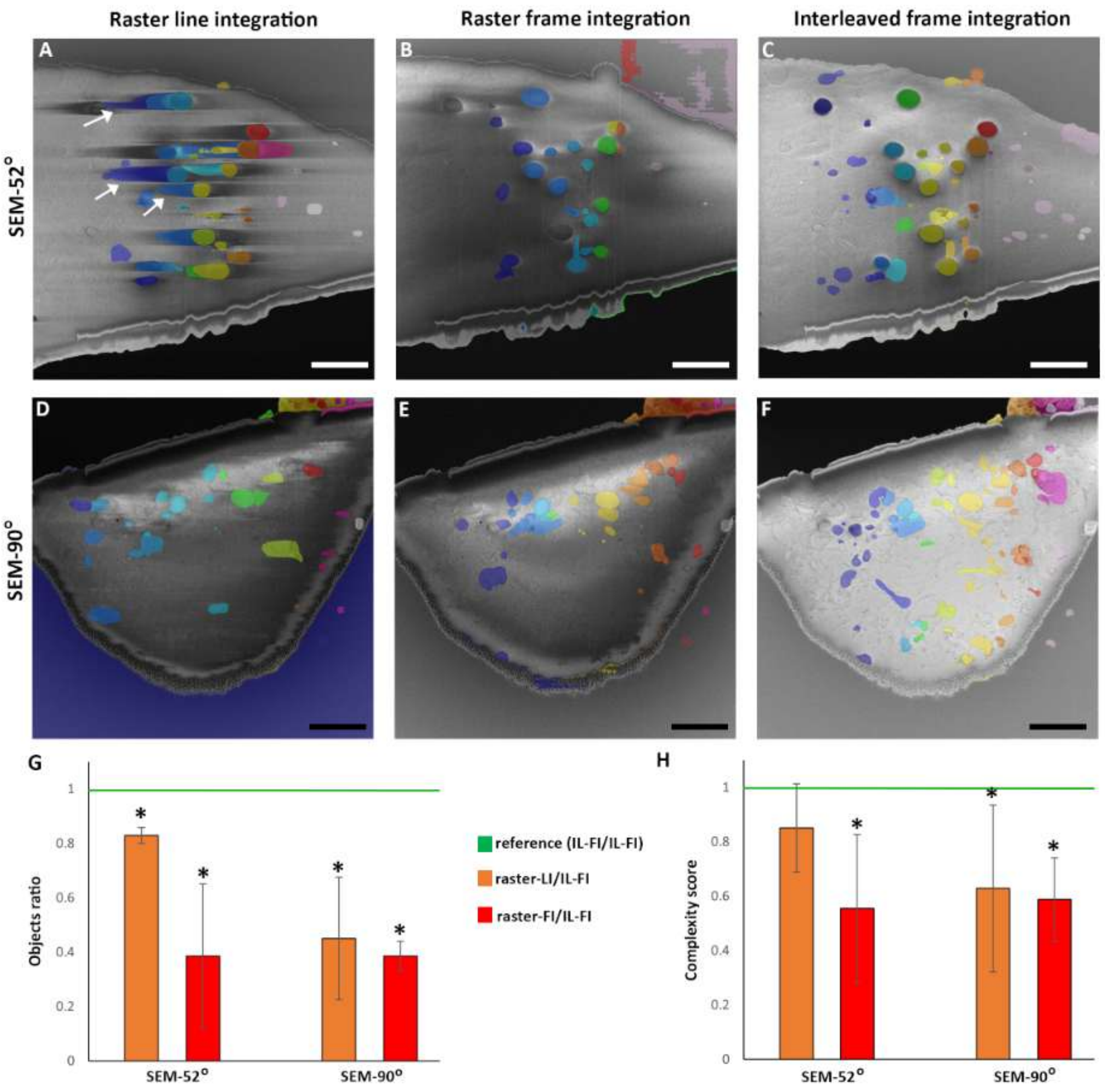

Supplementary Figure 8: Effect of scanning strategy on segmentation outputs.

Vitrified RPE-1 cells imaged at 52° (A to C) and 90° (D to F) with respect to the FIB milled sample plane using 100 ns dwell time x100 repetitions, raster line integration (A, D), raster frame integration (B, E) and interleaved frame integration (C, F). (A-F) SEM images (grey) overlaid with segmented areas (rainbow). White arrows indicate segmentation outputs at locations corresponding to charging artefacts. Scale bar: 2 µm. (G and H) analysis of vitrified mouse brain, RPE-1 and *E.gracilis* samples, * indicate statistical differences ($p<5\%$, $n=4$ per conditions). Ratio of number (G) or complexity (squared perimeter divided by the area) (H) of objects detected using the Segment Anything Model. Parameters were normalized for all datasets. The number of objects picked does not include objects which were not biological features. For example, where a picked object is entirely composed of charging artefacts it was not curated. Values obtained from raster line integration (LI) or raster frame integration (FI) acquisitions were compared to interleaved (IL) frame integration acquisitions.

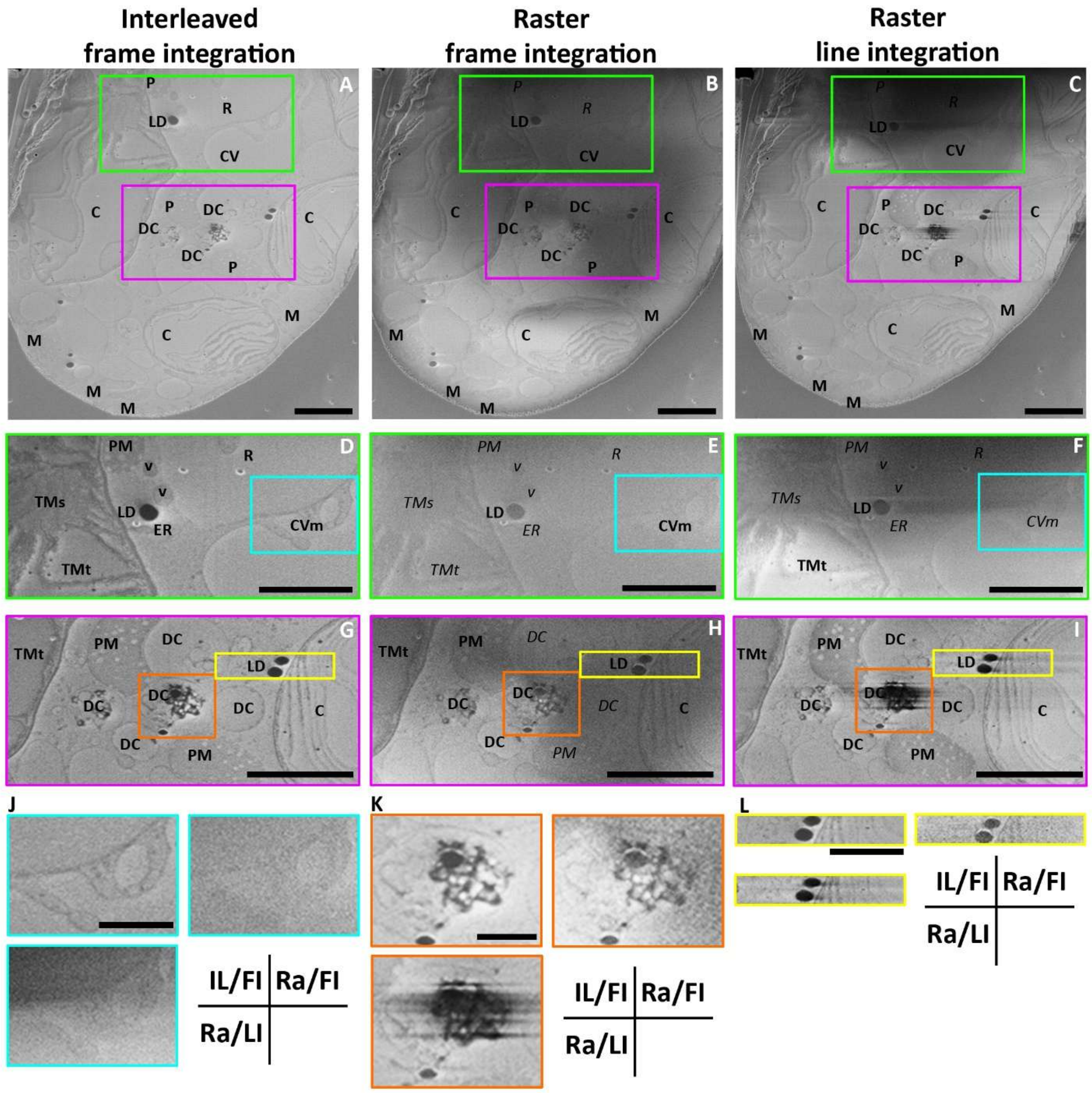

Supplementary Figure 9: Charging artefact mitigation in vitrified *E.gracilis* allows the observation of different vacuoles and degradation compartments.

Images of *E.gracilis* cells at 52° with respect to the FIB milled sample plane, 100 ns dwell time x100 repetitions using (A) raster line integration (Ra/LI) (B) raster frame integration (Ra/FI) (C) interleaved frame integration (IL/FI). P: paramylon, R: reservoir, CV(m): contractile vacuole (membrane), LD: lipid droplet, C: chloroplast, DC: degradative compartment, M: mitochondria, v: vesicles, ER: endoplasmic reticulum, TMs/t: thylakoid membrane sheet/tubes. Labels in italic indicate structures that were not observed for certain scanning strategies. (D to L) color-coded highlighted areas. Interleaved frame integration allows identification of compartments otherwise hidden by charging artefacts. (D-L) Brightness and contrast were optimised for visualisation. (D to F), enlarged areas showing the location of the contractile vacuole and the reservoir, panel in J shows increased contrast for IL/FI. (G to I and K) highlights of the modified scanning strategy enables observation of the content and the contact points of the degradation vesicle which are otherwise partially/totally obscured. (L) highlights that the presence of putative lipid droplets is not associated with the presence of charging artefacts allowing the observation of these compartments and their immediate surroundings. A-I scale bars: 2 µm, J-K scale bars: 1 µm.

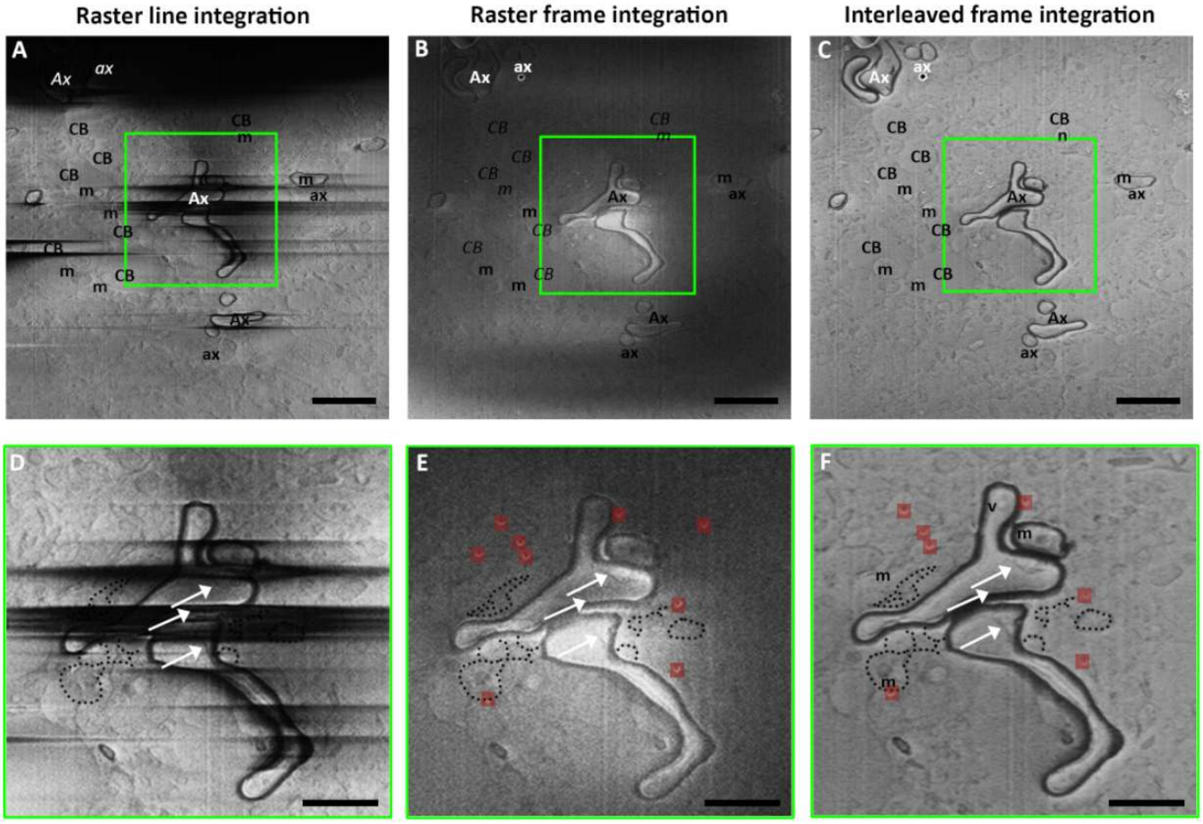

Supplementary Figure 10: Charge mitigation in SEM imaging of mouse brain tissue allows imaging of different myelinization thickness and axon content.

A 118-day old mouse brain imaged at 52° with respect to the FIB milled sample plane using 100 ns dwell time x100 repetitions. Brightness and contrast were optimised for visualisation. (A-C) CB: cellular body, Ax: thick myelin, ax: thin myelin. Italics indicate that the feature cannot be observed. Scale bars: 1 µm. (D-F) enlargement of (A-C) green box. The inner tongue of the oligodendrocyte (white arrow) is only visible in the image recorded using interleaved scanning. Outside the axon, mitochondria (m) and other cellular membranes are observed. In E and F red translucent rectangles highlight the location of burn spots. Scale bars: 500 nm.

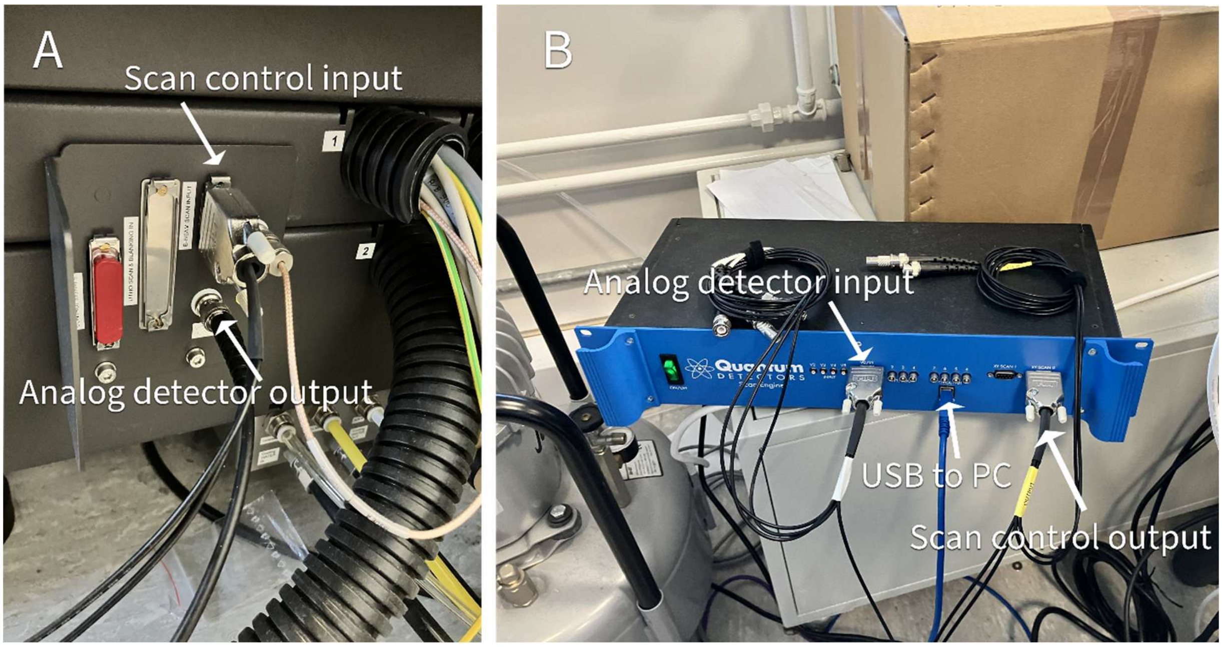

Supplementary Figure 11: Connections between the external scan control board of the microscope and the external scan engine. (A) scan control input and analog detector output of the microscope. (B) external scan control unit showing the scan control output, analog detector input and USB connection to a PC.

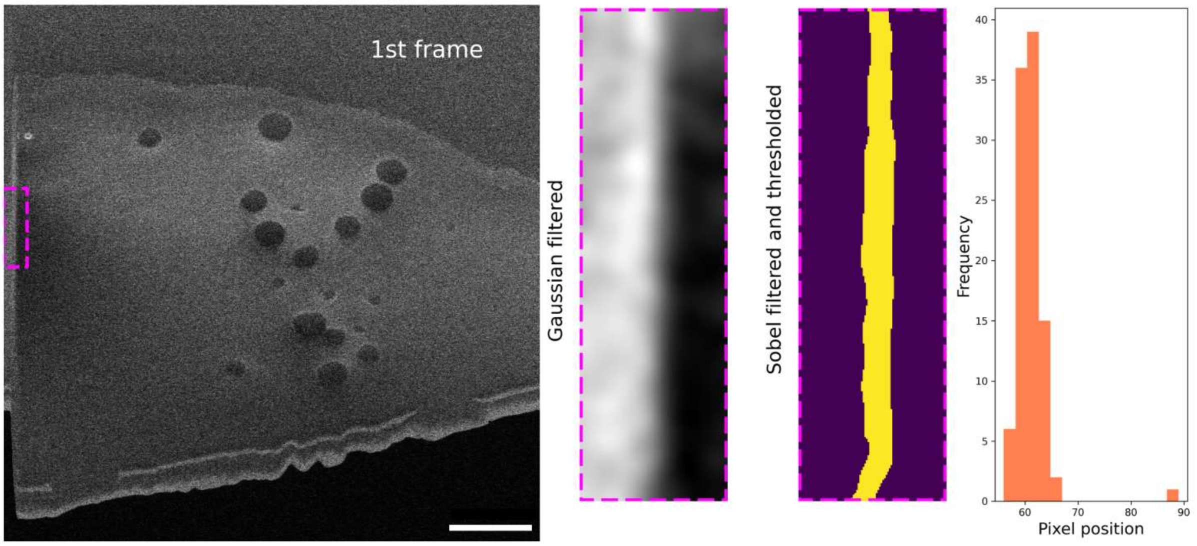

Supplementary Figure 12: Estimating the spatial extent of flyback distortions.

Processing of images from the vitrified RPE-1 sample, acquired using an interleaved scan, 100 ns dwell time x100 repetitions. Area showing distortions is indicated by a colored box in the image on the left. The same area was processed for all the frames as described in the text. Scale bar: 2 µm.

		Raster-line integration			Raster-frame integration			Interleaved-frame integration		
		100x100	500x20	10x1000	100x100	500x20	10x1000	100x100	500x20	10x1000
Raster-line integration	100x100		61.7%	15.9%	90.8%	18.1%	10.7%	2.4%	90.2%	89.6%
	500x20			22.9%	54.7%	10.3%	6.7%	9.5%	52.7%	58.6%
	10x1000				13.7%	34.7%	1.8%	57.1%	12.7%	17.4%
Raster-frame integration	100x100					21.5%	13.1%	1.9%	99.9%	97.0%
	500x20						78.0%	0.1%	18.2%	34.7%
	10x1000							0.1%	10.1%	25.4%
Interleaved-frame integration	100x100								1.4%	4.1%
	500x20									96.9%
	10x1000									

Supplementary Table 1: T test analysis of different samples imaged using different scanning strategies and fluence. Green values represent statistical differences between the populations (2 tails, difference variance).

		Raster-line integration			Raster-frame integration			Interleaved-frame integration		
		100x100	500x20	10x1000	100x100	500x20	10x1000	100x100	500x20	10x1000
Raster-line integration	100x100		51.3%	44.8%	6.2%	31.8%	29.6%	2.6%	22.8%	38.8%
	500x20			89.8%	85.0%	85.1%	80.5%	36.9%	95.0%	93.3%
	10x1000				98.9%	96.6%	92.0%	47.6%	92.4%	81.9%
Raster-frame integration	100x100					96.4%	89.6%	20.2%	82.8%	66.6%
	500x20						94.5%	41.6%	86.1%	74.0%
	10x1000							46.9%	80.1%	68.9%
Interleaved-frame integration	100x100								21.2%	18.3%
	500x20									83.3%
	10x1000									

Supplementary Table 2: T test of different samples imaged using varied scanning strategy and fluence. Green values represent statistically significant differences between the population (2 tails, difference variance).
