# Supplementary material for: Reduction of SEM charging artefacts in native cryogenic biological samples
Source: Nat Commun. 2025 Jun 4;16:5204. doi: 10.1038/s41467-025-60545-3 (PMC12137866; doi:10.1038/s41467-025-60545-3)
Supplement: Supplementary file 2 — Description of Additional Supplementary Files [file 41467_2025_60545_MOESM2_ESM.pdf]

Description of Additional Supplementary Files

Supplementary Movie 1: Volume acquisition of vitrified *E.gracilis*. Vitrified *E.gracilis* imaged at 52° with respect to the FIB milled sample plane using 100 ns dwell time x100 repetitions. A 1600 µm³ volume in focus was aligned and manually segmented for the region of interest.

Supplementary Movie 2: Volume acquisition of vitrified mouse brain cortex. A 118-day old mice corte imaged at 90° with respect to the FIB milled sample plane using 100 ns dwell time x100 repetitions. A 1334 µm³ volume in focus was aligned and manually segmented for the region of interest.
